# Policing extremism on gaming-adjacent platforms: awful but lawful?

**DOI:** 10.3389/fpsyg.2025.1537460

**Published:** 2025-07-31

**Authors:** William Allchorn, Elisa Orofino

**Affiliations:** International Policing and Public Protection Research Institute, Anglia Ruskin University, Chelmsford, United Kingdom

**Keywords:** extremism, gaming, policing, content moderation, P/CVE

## Abstract

Since the inception of video games, extremist groups have been able to create, modify, and weaponise this medium for activism and propaganda. More recently, the emergence of gaming-adjacent platforms (most notably Discord, Twitch, and Steam) has provided a key organizational infrastructure for recruitment and community building. This development poses a significant challenge for policing communities worldwide, particularly have been grappling with, especially in regard to the potential for extremist content to given the potential for extremist content on these platforms to contribute to radicalization and political violence. This article explores how policing communities are responding to extremist activity on gaming-adjacent platforms, the strategies they employ, and the effect these approaches have on extremist activism both online and, more crucially, offline. Using semi-structured interviews with 13 leading P/CVE practitioners, academic and technology industry experts, and content moderation teams, the article finds that third-party policing communities are adopting increasingly sophisticated tactics to counter extremist content. However, these efforts are increasingly undermined by the networked and adaptive nature of extremism, as well as by insufficient enforcement mechanisms at the platform level. In the future, this research suggests that fostering greater transparency in terms of service enforcement from above, combined with efforts to counter toxic and extremist-“adjacent” cultures from below, may enhance resilience against the spread of extremism on gaming-adjacent platforms.

## 1 Introduction

Gaming is one of the most popular leisure activities of our time. More people than ever before are playing games and congregating in gaming-related digital spaces. An estimated 3.32 billion—approximately one-third of the world's population—play video games, and forecasts suggest that this number will continue to rise in the coming years (Statista, 2025). Currently, millions of fans fill esports arenas (some as big as soccer stadiums), while even more engage on gaming-adjacent platforms such as Steam, Discord, Twitch, or DLive to discuss gaming, stay informed about their favorite video games, watch livestreams of popular gaming influencers, and connect with other players. In fact, in the first quarter of 2025 alone, Twitch^1^ recorded 240 million monthly visitors. Unsurprisingly, the booming gaming industry is expected to continue its rapid growth in both size and revenue (World Economic Forum, 2022; Statista, 2025).

In accordance with the growing popularity of gaming activities across the world, research on video games, gaming-related content, and digital gaming spaces has also been gaining momentum, steadily increasing since the 1990s (Kowert and Quandt, 2016). Over the last few decades, a substantial body of research has accumulated on the social and psychological appeal of games, gaming communities, and gamification, as well as the potential negative and positive effects of gaming and related activities (see Hodent, 2021, for an overview). However, until recently, research on the alleged negative impact of playing video games has largely focused on and controversially discussed a potential link between gaming and aggression or gaming addiction. However, a new key area of concern has recently come to the center of attention: a potential connection between gaming and extremism.

The study of the intersection between gaming and extremism has focused largely on a few key areas: the presence of gamers and gaming culture in extremist communities, with a particular focus on the gamification of extremism (Schlegel, 2020; Lakhani and Wiedlitzka, 2023); the co-option of gaming aesthetics and culture by extremists (Munn, 2019); the creation of (mods and bespoke) games by extremists (Robinson and Whittaker, 2021); and the potential vulnerabilities that might make gamers more susceptible to radicalization than the general population (Condis, 2020).

However, although a growing body of literature exists on the topic, one area that requires more thorough investigation is gaming-adjacent platforms, and, more specifically for this current study, the policing of extremist (borderline) content on these platforms. These are digital platforms that were originally created to support the broader gaming community online, either by facilitating community building and conversation between gamers or allowing gamers to livestream their activity.

On the one hand (and as outlined by Davey, 2024, p. 95), they can be seen as the “digital infrastructure that surrounds gaming, a cornerstone of global gaming communities, and essential to the transmission of gaming culture.” On the other hand, gaming-adjacent platforms (namely Steam,^2^ Twitch, and Discord^3^) have gained notoriety due to their ability to be seen as digital playgrounds for extremists to exploit (Schlegel and Kowert, 2024).

This is starkly illustrated by several high-profile cases of extremist use of gaming-adjacent platforms. In recent years, there have been a number of incidents of extremist activity on Discord. Perhaps most notoriously, the white supremacist attacker who killed ten in Buffalo, New York (USA) in May 2022, used a private Discord server as he planned his attack, sharing his diary and manifesto with friends on the platform (Thompson et al., 2022). The platform was similarly used by the planners of the 2017 Unite the Right rally, which saw hundreds of right-wing extremists gather in Charlottesville, Virginia (USA). It culminated in a vehicular attack on counter-protesters that left one individual, Heather Heyer, dead, and 35 others injured (Davey and Ebner, 2017).

Additionally, German far-right extremists utilized the platform to coordinate efforts to disrupt the 2017 German federal election through targeted harassment and the use of “meme warfare”—the targeted spamming of ideological content in the form of memes with the intention of injecting extremist talking points into broader discussion around the election (Davey and Ebner, 2017). Furthermore, livestreaming platforms have been utilized by extremists on multiple occasions over the last 5 years. Twitch was used to broadcast a 2019 attack on a synagogue in Halle (Germany), which left two people dead (Wong, 2019), as well as the 2022 attack in Buffalo (Hern and Milmo, 2022). DLive was similarly used by several users to livestream the 2021 insurrection at the US Capitol (Hayden, 2021).^4^

The above examples illustrate the concerning use of gaming-adjacent platforms by extremists and, in and of themselves, demonstrate the importance of analyzing these platforms to those who wish to understand and counter extremists' digital strategies. The urgency of this need is compounded by the growth in popularity of these platforms. Both Discord and Twitch are experiencing steady increases in their user bases, driven in part by strategies designed to broaden the user base of these platforms beyond gamers. This expansion provides an opportunity for established extremist communities to radicalize and reach new audiences. This is of particular concern when the central role of online communications in radicalization is considered—between 2010 and 2020, the Profiles of Individual Radicalization in the US (PIRUS) dataset, the largest database of open-source information on radicalized individuals in the USA, showed a 413% rise in the internet playing the primary role in the radicalization process for those under the age of 30 compared to the previous decade (Hitchens and Ayad, 2023, p. 6). Accordingly, analyzing gaming-adjacent platforms provides the opportunity to better understand the ways in which extremists engage in gaming and gaming culture, and also a potential window into how extremist content can be better policed online.

In this study, we aim to address this lacuna by answering the following research questions:

1- What expressions of hatred and extremism have been noted by practitioners and content moderators on gaming-adjacent platforms?
2- What approaches are adopted by content moderators toward extremism vs. terrorism on gaming-adjacent platforms?
3- What Preventing and Countering Violent Extremism strategies are being employed?

## 2 Literature review: the role of extremism on gaming-adjacent platforms

### 2.1 Emerging field and institutional foundations

The literature on extremism in gaming-adjacent platforms is still nascent, marked by exploratory studies and institutionally driven reports. Key foundational work has been undertaken by organizations such as the Institute for Strategic Dialogue (ISD) and the Anti-Defamation League (ADL). ISD's longitudinal research (Davey, 2021, 2024; Thomas, 2021) has mapped the presence of extremists across various platforms, including DLive, Twitch, Steam, and Discord. Complementarily, the Anti-Defamation League (2020, 2022, 2023) has explored systemic harassment, moderation challenges, and the structural gaps faced by trust and safety professionals. Additionally, broader institutional actors, such as the Radicalization Awareness Network (Lakhani, 2021) and the United Nations Office of Counter-Terrorism (Schlegel and Amarasingam, 2022), have provided overviews from a practitioner's standpoint. However, these efforts are often more descriptive than analytical, highlighting the need for a deeper theoretical and conceptual base.

### 2.2 Platform affordances and radicalization pathways

Recent work has begun to explore the specific mechanisms and affordances of gaming-adjacent platforms that facilitate radicalization. Davey's (2024) ethnographic study of 45 Steam groups and 24 Discord servers identifies these spaces as echo chambers and recruitment pipelines, where demographic distinctions (e.g., younger users on Discord vs. older on Steam) may demand platform-specific moderation strategies. However, the study stops short of critically evaluating the role of platform design and governance in shaping user resilience or vulnerability to extremism. Similarly, Koehler et al. (2023) point to the ideological proximity forged through ambient exposure, suggesting a shift from targeted recruitment to more affective, cultural immersion-based models of radicalization. This represents a theoretical advancement by challenging older paradigms focused narrowly on direct interpersonal grooming.

### 2.3 Cultural and symbolic appropriation in gaming spaces

An important theme across the literature is how gaming culture itself is appropriated for ideological ends. Moonshot's (2024) content analysis of platforms, including Discord, 4chan, Gamer Uprising, and incels.is uncovers the use of game lore and symbolism to validate extremist worldviews. Their findings open critical lines of inquiry into the semiotic infrastructure of games—character archetypes, in-game hierarchies, and narratives—and how these can reinforce far-right ideologies around masculinity, order, and societal decline. This symbolic appropriation highlights a broader trend: gaming spaces are not merely neutral backgrounds for extremist activity, but active cultural fields where ideology is coded into shared language, imagery, and rituals.

### 2.4 Quantitative insights and limitations

Though qualitative work dominates the field, there are growing efforts to provide quantitative data. Surveys by Kowert et al. (2024) and Winkler et al. (2024) reveal that while extremist behavior is often encountered directly within games, a significant portion occurs in adjacent spaces such as forums, chat servers, and modding communities. These insights underscore the need to treat the gaming ecosystem holistically, rather than isolating games from their social and technological contexts. However, survey-based methodologies face limitations such as self-reporting bias and often lack the granularity to explain how and why such interactions escalate into deeper radicalization processes.

### 2.5 Governance, moderation, and platform accountability

Several studies critique the regulatory and governance failures of platforms in addressing extremist content. Winkler et al. (2024), for example, provide disturbing evidence of the glorification of Nazism and Islamist propaganda on Discord and Roblox. They highlight the commercial and algorithmic logics that allow such content to persist, raising urgent questions about platform incentives, regulatory pressure, and community backlash. Despite this, there remains a theoretical gap around the political economy of moderation—how platform decisions are shaped not only by technical capabilities but also by broader market and political forces.

### 2.6 Representational politics and media framing

Finally, Collison-Randall et al. (2024) introduce a novel perspective through media framing analysis. Their study of 81 news articles across 10 countries categorizes public narratives into informative, provocative, derogatory, and policy-oriented frames. This work underscores the importance of discursive representation—how media shapes societal understandings of gaming and extremism, often in reductive or sensationalist ways. Though less focused on platform dynamics, this framing adds nuance to the debate by situating public perception as a key mediating factor in policy and platform responses.

### 2.7 Summary

Whilst numerous articles and reports provide recommendations, there is less focus on how such extremist sentiments are actively policed or on the direction platforms are taking in relation to content moderation. An exception is the ADL's (2023) study, which highlights the challenges faced by trust and safety employees in the gaming industry when moderating hate and harassment. However, the majority of existing studies tend to privilege passive, ethnographic, or survey-based approaches over engaged, in-depth, semi-structured interviews. This study aims to address that gap by drawing on interviews with content moderators, P/CVE practitioners, academic and technology industry experts who are on the front lines of combatting radicalization, both online and offline, to answer the following research questions:

1. What expressions of hatred and extremism have been noted by practitioners and content moderators on gaming-adjacent platforms?
2. What approaches are adopted by content moderators toward extremism vs. terrorism on gaming-adjacent platforms?
3. What strategies are being employed to prevent and counter violent extremism?

## 3 Materials and methods

This study adopted a qualitative research design to examine expressions of extremism, content moderation practices, and Preventing and Countering Violent Extremism (P/CVE) strategies on gaming-adjacent platforms. Between May and July 2024, semi-structured interviews were conducted with 13 participants, including content moderators, P/CVE practitioners, academic and tech industry researchers who had direct, professional experience with gaming-adjacent platforms (such as Steam, Twitch, and Discord) [this interview-based research was complemented by a qualitative document analysis of platform Terms of Service and a systematic literature review of academic research on extremism in gaming-related contexts (though the findings of the literature review are not presented here)].

The study was grounded in a constructivist epistemology (Liu and Matthews, 2005; Winner, 1993a,b), emphasizing how practitioners make sense of and respond to the challenges of content moderation and extremism. The approach allowed the research team to explore how knowledge and practice are shaped by individual experiences, organizational policies, and platform governance structures. Grounded in a constructivist epistemology (Liu and Matthews, 2005; Winner, 1993a,b) and informed by a sociological framework of policing (Loader and Mulcahy, 2003; Reiner, 2010), the study examined how authority, discretion, and legitimacy are negotiated in digital environments. This theoretical orientation shaped both how data were gathered—focusing on practitioner perspectives—and how they were analyzed, emphasizing contextual nuance, institutional norms, and community dynamics.

### 3.1 Participants

As noted in Table 1 below, thirteen individuals participated in the study between May and July 2024. Participants included content moderators, Preventing and Countering Violent Extremism (P/CVE) practitioners, academic researchers, and industry professionals with direct experience of gaming-adjacent platforms such as Steam, Twitch, and Discord.

**Table 1:** Demographic profile of interviewees.

**Demographic**	**Number**	**Percentage %**
**Gender**		
Male	8	61.5384615
Female	5	38.4615385
Total	13	100
**Location**		
UK	9	69.2307692
US	1	7.69230769
Germany	2	15.3846154
Total	13	100
**Profession**		
Content moderators	3	23.0769231
P/CVE practitioners	4	30.7692308
Academic experts	2	15.3846154
Tech industry experts	4	30.7692308
Total	13	100

Participants were recruited using a combination of convenience and snowball sampling methods (Noy, 2008), with a focus on professional diversity, gender, and geography. Initial contacts were drawn from the research team's professional networks, and subsequent participants were referred by these individuals. Efforts were made to ensure diversity in professional roles, gender, and geographical representation, though there was a slight skew toward UK-based male respondents. While there was some UK-based male skew, the sample included a range of stakeholder perspectives critical to understanding the relational and discretionary aspects of moderation—key concerns of a policing-informed approach.

### 3.2 Materials

The primary data collection tool was a semi-structured interview schedule, developed in accordance with best practices (Kallio et al., 2016). The design process involved three key stages:

- Derivation from research questions: initial themes and prompts were directly aligned with the study's core research questions (Turner, 2010):
  ○ What expressions of hatred and extremism have been noted by practitioners and content moderators on gaming-adjacent platforms?
  ○ What approaches are adopted by content moderators toward extremism vs. terrorism on gaming-adjacent platforms?
  ○ What P/CVE strategies are being employed?
- Literature review integration: relevant academic and gray literature informed the development of the script (e.g., Conway et al., 2019; Ahmed et al., 2020), ensuring coverage of underexplored themes.
- Iterative review and piloting: the interview guide was refined through internal piloting to ensure clarity, neutrality, and comprehensiveness (Patton, 2015).

The final schedule included background questions on participants' roles, followed by prompts on:

- Experiences with extremist or hateful content
- Moderation practices, including bans and cooperation with law enforcement
- Reflections on current P/CVE interventions, challenges, and ethics

Interviewers used probes flexibly to allow for deeper or emergent insights beyond the structured prompts. The interview schedule was designed around the study's research questions, refined through internal piloting (Patton, 2015), and shaped by both existing literature (e.g., Conway et al., 2019; Ahmed et al., 2020) and a sociological framework of policing. The latter emphasized exploring how moderators and practitioners define and enforce boundaries around extremist content, how discretion is applied, and how legitimacy and trust are maintained or challenged in online communities. Questions probed experiences with extremist or hateful content, decision-making processes, enforcement strategies, and reflections on the ethics and impacts of moderation.

### 3.3 Procedure

#### 3.3.1 Data collection

Data were collected via in-depth semi-structured interviews. This approach enabled exploration of participants' lived experiences and professional perspectives in detail (Leech, 2002). Interviews were conducted remotely, audio-recorded with consent, and transcribed verbatim. This method allowed for rich, situated insights into how moderation is enacted as a form of social control—analogous to street-level policing—where discretion, ambiguity, and community relations are key.

#### 3.3.2 Data analysis

Interview transcripts were analyzed using reflexive thematic analysis, following Braun and Clarke's six-phase framework (Braun and Clarke, 2006, 2021; Clarke and Braun, 2017):

1. Familiarization with the data
2. Generating initial codes
3. Searching for themes
4. Reviewing themes
5. Defining and naming themes
6. Producing the report

Coding was primarily semantic, staying close to participants' language and context (Braun and Clarke, 2022a,b). The sociology of policing lens informed theme development by directing attention to power dynamics, enforcement discretion, institutional norms, and community perceptions. For instance, particular analytic emphasis was placed on how moderation decisions were framed by cultural expectations, platform affordances, and the pressures of both community and corporate stakeholders. All transcripts were analyzed as a single data corpus, enabling synthesis across roles and contexts.

One researcher conducted the initial coding in NVivo (QSR International Pty Ltd., 2020), with a second researcher cross-checking codes and assisting in refining themes. The research team collaboratively interpreted the findings, grounding them in a constructivist epistemology (Liu and Matthews, 2005; Winner, 1993a,b), which emphasized how practitioner knowledge is shaped by organizational and platform-specific contexts.

### 3.4 Ethics statement

#### 3.4.1 Human subject research

The studies involving humans were approved by the Anglia Ruskin University Ethics Committee. The studies were conducted in accordance with the local legislation and institutional requirements. The participants provided their written informed consent to participate in this study.

#### 3.4.2 Ethics

The study was approved by the relevant institutional ethics board and conducted in accordance with qualitative research ethics guidelines (British Psychological Society, 2021; Wiles, 2013). Participation was voluntary, and interviewees could decline to answer any questions or withdraw at any stage.

To minimize ethical risks:

- Interview prompts were worded carefully to avoid distressing language.
- Participants were encouraged to guide the depth and direction of their contributions.
- All data were anonymised and stored securely in compliance with GDPR and institutional protocols.

The research team maintained reflexivity throughout, acknowledging the power dynamics between researchers and participants, as well as the potential sensitivities surrounding professional roles and confidential content. This integrated theoretical and methodological approach supported a context-sensitive analysis that recognized moderation not just as rule enforcement but as a negotiated, relational process involving legitimacy, discretion, and community resilience—core concerns in both policing and platform governance.

## 4 Results

### 4.1 Breakdown of themes and sub-themes

Initial coded data were reviewed and analyzed to determine how different codes may be combined according to shared meanings, allowing them to form themes or sub-themes.

**Figure 1:**
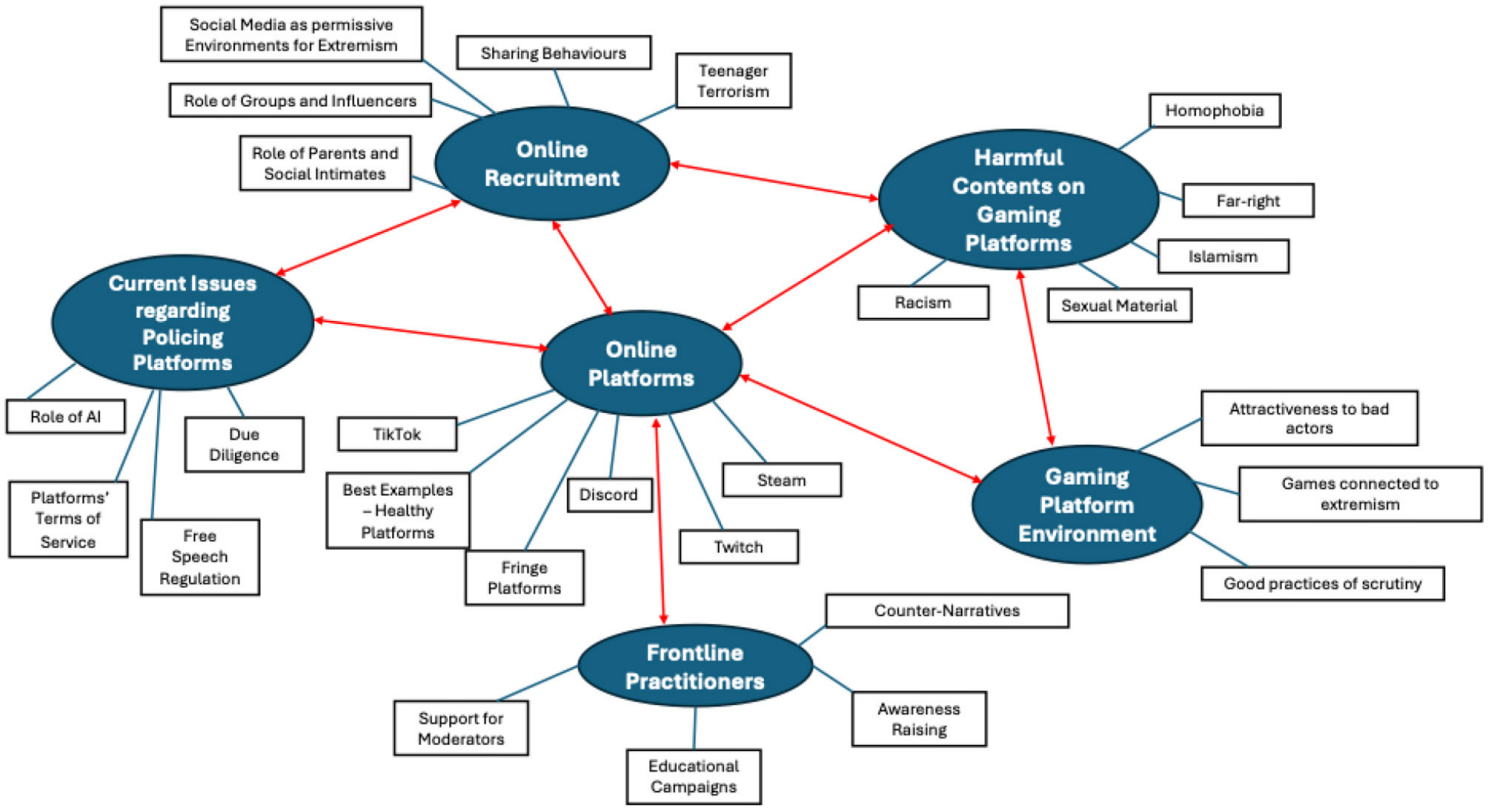
Initial thematic map.

Figure 1 provides a visual representation of the initial six main themes and 27 sub-themes identified, which are representative of the overarching narratives within the data, as identified by the researchers through various iterations (Braun and Clarke, 2012).

As shown by the red connectors in Figure 1, the main themes appear to be all intertwined with the central theme “online platforms,” as the focus of this research project. In relation to online platforms—specifically gaming-adjacent spaces—participants discussed “online recruitment” strategies of extremist actors (both groups and solo), the kinds of extreme and “harmful content” they have encountered most in their experience working on such platforms, and the “environment” characterizing gaming-adjacent platforms in terms of opportunities for malicious/extremist actors. Another main theme identified was connected to “issues regarding policing online platforms,” specifically related to the monitoring mechanisms already in place on various platforms, including the use of AI and the terms of service. Participants often highlighted how policing mechanisms can conflict with free speech regulations and contentious debates surrounding censorship.

Finally, overarching narratives surrounding “frontline practitioners” were regrouped into a main theme pertaining to what such practitioners can do to improve online safety against extremism in gaming spaces and their need for more support. This initial thematic map was then revised in light of the main research questions of this research:

1. What expressions of hatred and extremism have been noted by practitioners and content moderators on gaming-adjacent platforms?
2. What approaches are adopted by content moderators toward extremism vs. terrorism on gaming-adjacent platforms?
3. What strategies are being employed to prevent and counter violent extremism?

They were then reorganized in the final thematic structure outlined in the result section below. In the transition from the initial thematic structure to the final one, a variety of sub-themes were identified but discarded for this project as not fitting with the research questions (Braun and Clarke, 2012).

**Figure 2:**
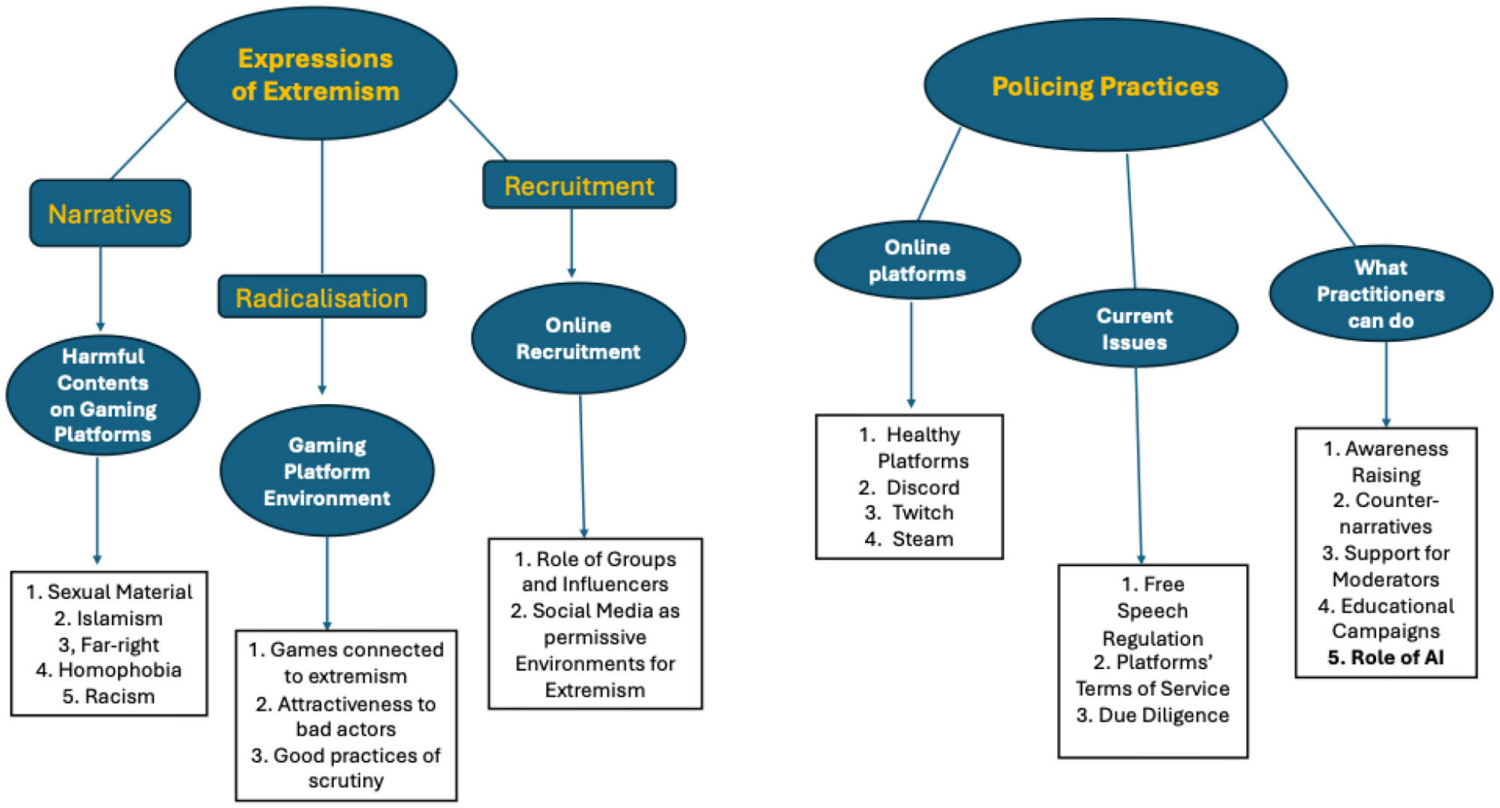
Final thematic structure.

As highlighted in Figure 2, the six themes identified were reorganized in the final thematic structure into two macro-themes: “expressions of extremism” and “policing practices,” each of which is extensively discussed in the section below.

Within “expressions of extremism,” the research team felt the need to further differentiate the sub-themes by adding another layer—clustering the topics in the following thematic groups: “narratives,” “radicalization,” and “recruitment.” These thematic groups (aka second-level sub-themes) then include three of the sub-themes identified in the initial thematic structure, i.e. “harmful content on gaming platforms,” “gaming platform environment,” and “online recruitment” (see Figure 1) and their related child themes (fourth-level sub-themes).

Regarding the second macro-theme (i.e. “policing practices”), the research team did not see the need to add any further layer of sub-themes. Hence, this macro-theme articulates in three levels, including the three remaining main themes identified in the initial thematic structure (see again Figure 1), i.e. “online platforms,” “current issues,” and “what practitioners can do,” and their child themes (third-level sub-themes).

By comparing Figures 1, 2, it emerges that the sub-theme “role of AI” (highlighted in bold in Figure 2) was included as a child-theme of the second-level theme “what practitioners can do.” This decision was made as interviewees extensively discussed how AI could effectively contribute to better moderation efforts. Finally, the only sub-themes discarded for this project were “role of parents and social intimates,” “teenager terrorism,” “sharing behaviors,” “TikTok,” and “fringe platforms.” These themes were not extensively discussed by the participants in light of the research questions and interview protocol. However, the research team acknowledges their value and will consider expanding on them in future studies related to extremism and gaming.

### 4.2. Expressions of extremism

#### 4.2.1 Narratives

Among the respondents, extremist ideologies that were most frequently mentioned as manifesting on gaming-adjacent platforms appeared through a spectrum of ideologies, each with its own distinct themes and messages. The most prevalent ideology was right-wing extremism, which saw a range of expressions on these platforms rotating around sentiments related to white supremacy, Nazism, and anti-Semitism, often marked by explicit expressions of racism, misogyny, homophobia, and conspiracy theories, like QAnon:

“We found a lot of right-wing extremism and white supremacy, anti-Semitism, racism, misogyny and like really hateful misogyny too. Not just sort of all women are stupid gamers, but like, you know, a level beyond that. We also found a lot of conspiracy content related to the sort of the great replacement, but also Q Anon and that type of thing.” (Interviewee 12, July 2024)^5^

On the other side, Islamist extremism was less remarked upon, but practitioners and moderators here saw ideals promoted on gaming adjacent platforms such as the establishment of a Caliphate, the call for Jihad, and loyalty to the Ummah, or global Muslim community.

“They're sharing IQB [Izz al-Din al-Qassam] videos of terrorist propaganda and regardless of the ethics of the conflict, like it's legal status in the UK, as a terrorist organization, it is illegal to share this content. And yet you can find it anywhere.” (Interviewee 11, June 2024)^6^

Additionally, and quite pertinently, interviewees noted narratives and online content on gaming-adjacent platforms cleaving to ‘extremist-adjacent' fixations and egregious content that most definitely broke platform terms of service, such as child sexual abuse materials (CSAM), fixation on school shootings, and graphic depictions of sexual content and violence:

“There is a lot of content that's uploaded that definitely glorifies mass shootings. That and that have very weirdly specific rules, like if they're just uploading a map of Columbine High School, then it's OK, or Sandy Hook, then it's OK. They have to be very explicit about supporting it.” (Interviewee 4, June 2024)^7^

“I've seen like from graphic violence to hate speech to Homophobia. Sinophobia, like every kind of hate that you can imagine. I've seen it's also like the sexual stuff, like we see that a lot on the platform. It's a lot of like some reason that fixed station about sexual abuse and stuff.” (Interviewee 5, June 2024)^8^

This latter point both echoes and goes beyond recent studies by Moonshot (2024) and Winkler et al. (2024) based on user surveys and content analysis of the prevalence of extremist sentiments in gaming-adjacent spaces to suggest that hybridized extremism is far more problematic than first thought on these platforms.

#### 4.2.2 Radicalization

We also found among respondents that, whilst these different extremist narratives were ideologically distinct, they reflect new forms of radicalization across the board that are fuelled by a new set of tactics mapped onto the gaming-adjacent ecosystem. In particular, interviewees found that radicalization itself occurred on such platforms through either organic or strategic means (Schlegel, 2021a,b). For example, some individuals are purposefully directed into these spaces through pre-existing extremist channels, while others actively seek them out on their own. As one interviewee suggested, “You only end up in that space if you're seeking that out, and if you've been channeled to it through already pre-existing extremist channels” (Interviewee 6, May 2024).^9^

Adding to this strategic and interactive set of processes, radicalization within the gaming-adjacent platform ecosystem was also found by respondents to be self-directed and post-organizational, with individuals adopting symbols and rhetoric to identify with broader ideological trends rather than joining a specific group. As one interviewee noted, “It's very decentralized, and people reappropriate various historical symbols to self-identify more with the genre of ideology than a specific group” (Interviewee 2, June 2024).^10^

Another significant component of radicalization on gaming-adjacent platforms, used in a manner similar to more formal extremist groups, is a process known as content funneling. For instance, initial interactions may take place in online gaming communities where individuals bond over shared interests (Williamson, 2020). These interactions can then migrate to less-regulated gaming-adjacent platforms, allowing extremist rhetoric to proliferate unchecked. As one interviewee pointed out:

“Direct sort of grooming or cultivation might start on games. That's where you have matchmaking. It's where you can build quick rapport with people. But that's the stuff that very quickly moves to adjacent platforms, where there's sort of less monitoring.” (Interviewee 8, June 2024)^11^

Both of these tactics and trends were observed by Davey (2024) in his digital ethnographic study of right-wing extremist communities on Discord and Steam, showing how gaming-adjacent platforms can act as a bridge between more popular platforms and more explicit and egregious closed spaces.

#### 4.2.3 Recruitment

Connected to this radicalization pipeline (Munn, 2019) were several other, softer tributaries [or streams (Munson, 2002)] into extremist subcultures and milieux on gaming-adjacent platforms that were detected by respondents. These included a pattern of idolisation, machismo, and community-building processes.

Respondents, for example, remarked on how high-profile figures such as the Norwegian attacker, Anders Breivik and the Christchurch attacker, Brenton Tarrant, were considered as “idols” for aspiring extremists (Lewis, Molloy and Macklin, May 2023), providing both models of action and a sense of belonging that was being further gamified and glorified on the platform: “Breivik's a big idol and also the Christchurch attack….” (Interviewee 3, May 2024).^12^ Additionally, and cleaving to the hybrid nature of extremism, the allure of a lawless environment, respondents also mentioned the hyper-masculine environment in games such as GTA V and Call of Duty that can be appealing for individuals attracted to extremist spaces: “finding people that are allured by that machismo and by that lack of rule of law is something that you could see as particularly appealing” (Interviewee 2, June 2024). ^13^

This often involves a pseudo-military team-building approach, where groups adopt survivalist and combat skills to unite against a shared adversary, typically the opponent of a particular extremist group (Al-Rawi, 2016). Indeed, and according to Schlegel (2021a; 2021b, p. 6–7), sustained engagement with the narratives propagated through such gaming-adjacent applications could, potentially, increase “susceptibility to radicalization” and legitimize further inroads into real-world offline violence. As another interviewee intimated, “It's almost transferring a digital version of those kinds of survivalist skills and team-building skills because you're working as a team together to kill a common enemy” (Interviewee 1, May 2024).^14^

### 4.3 Policing practices

When asked to reflect on current policing practices on gaming-adjacent platforms, participants primarily focused on three main sub-themes: (1) Healthy platforms; (2) Current issues, and (3) What practitioners can do. This section will discuss the findings of all three sub-themes in detail below.

#### 4.3.1 Healthy platforms—What they look like (ideally)

When it came to policing extremist content on gaming-adjacent platforms, respondents were asked first and foremost what was not happening at this point, and how healthy online platforms play a crucial role in counteracting extremist content. In this sense, respondents suggested that effective platforms would have swift content removal mechanisms, vetted content channels on their sites, and stricter scope for banning and removing the most persistent violators of the terms of service. Participants mentioned as examples of ideal responses Twitch's removal of the Buffalo shooting livestream within 2 min (Grayson, 2022), specific Facebook and Reddit subchannels where content is vetted before going live (Thach, 2022) and Discord's efforts (Discord, 2024) to unlist and remove servers exploited or set up by formally identified extremist organizations. Moreover, a “clear set of rules” users need to agree to before entering any platform was identified as good practice for a healthy digital environment. Participants highlighted that users should agree to

“a set of rules which are in line with the platform and the community guidelines, which are things like be kind, be supportive, no misogyny. When somebody joins, they should immediately go to the rules … so they can decide what they're interests are, but they have to agree to the rules before being able to [do] anything else on the platform” (Interviewee 13, May 2024).^15^

However, participants also complained that platforms often fail to enforce their community guidelines, thereby compromising their effectiveness.

When speaking about healthy platforms, participants also reiterated the fact that gaming-adjacent communities are generally healthy places of community building, where people find their preferred subcultural groups and create long-lasting social bonds through common interest, which might also not pertain exclusively to gaming.

“You have so many streams that have nothing to do with gaming, on sports, on politics, on just chatting, you know, influencers just talking to the camera.” (Interviewee 12, July 2024)^16^

Whilst acknowledging the positive community-building power of such digital environments, interviewees also believed that genuine and benign interests are exploited by extremist actors to disseminate their ideologies. One interviewee stressed the fact that extremist actors have multiple identities, and they take advantage of their multiple hats to add a layer of plausible deniability to their more illicit activities in digital spaces:

“…now we know very little about sort of the hobbies that extremists have, right? We're always talking about them as being extremists 24/7, but these are people with, you know, regular other interests as well. So, it could very well be that they just happen to congregate on these platforms and then play together and find their community there. And then we see these groups on gaming-adjacent platforms.” (Interviewee 12, July 2024)^17^

As interviewee 12 went on to mention, this makes it very hard to vet people as they infiltrate specific communities where they share interests before disseminating specific extreme ideas. While (some) platforms are making efforts to vet users better and make them agree to a specific set of rules (as outlined at the beginning of this section), participants also highlighted important issues on gaming-adjacent platforms that remain largely unaddressed and work as a gateway to extremist content within these digital environments.

#### 4.3.2 Current issues

Among the most pressing issues reported by the interviewees, comment moderation was highlighted. While posts are “easier” to be vetted, users' replies and comments are apparently not as straightforward for fear of backlash or potential blowback toward the platform. Furthermore, it is the language of the gaming ecosystem that also makes moderation quite hard, as terms that would receive “red flags” on other platforms (such as “kill,” “enemy,” and “eliminate”) are constantly used for gaming purposes. Moreover, it was learned from moderators that they are sanctioned to intervene in in-game chats: “We do not moderate like chats in games…it's only everything that is on the platform” (Interviewee 5, June 2024).^18^ So, unless gamers report something wrong in in-game chats, these environments tend to be quite unmoderated. Moderators themselves often expressed frustration with inconsistent enforcement policies and the burden of deciding whether to report content or users to local law enforcement agencies. Interviewees have reported how some channels appear to be very much unregulated, as:

“they offer a sort of open-door policy where people can just come in and say what they want because there are no steps there already….So I think for the majority of servers that will have problems with harassment and issues where they're more like at a prevalent is probably because they don't actually have rules in place or they don't have something even just like a soft guard to be able to say we don't accept this here.” (Interviewee 13, May 2024)^19^

Moderation efforts were also reported to be impacted by the use of hidden extremist symbols in communication styles and by the fact that these symbols appear to change quite often. An example reported by one interviewee is the hawk:

“The hawk also plays a big role. After the 7th of October, you can say on the one side that this is harmless. On the other side, it's a sign for the community. It's a code for the community, and it's also maybe even in some regards, a call for action.” (Interviewee 3, May 2024)^20^

Connected to the use of symbols and creativity, participants also stated that gaming communities appear as malign “creative outlets” to express extremist fantasies and dreamscapes:

“So you have the kind of in-game element where you can express how your extremist world views, and this is something that users do and talk about how they kill certain people in games and target them specifically because of their mindset. You have a broader audience that is exposed to this content to some extent because it's not a niche space people congregate in. And you have this kind of creative outlet of expressing your extremist world views rather than through sharing, like news articles, or just talking about what you believe in. You show that in the gaming space and in the game itself, if you can.” (Interviewee 6, May 2024)

Finally, one issue continuously raised by interviewees was cross-platform frustrations concerning younger users' access and coming under the influence of notorious extremist individuals (i.e. influencers) who were streaming live gameplay along with extremist narratives openly on mainstream social media and gaming-adjacent platforms. Yet, their accounts had not been taken down.

“We had a couple of instances where people were overheard by parents in some of the conversations over their headsets where even just listening to half of the conversation, the parents were quite concerned that there was radicalisation taking place…and in both instances that came my way, that was proven to be the case of the far right. We were made aware of instances usually by teachers where parents have flagged to the school that their kid had put the client tag like ‘all coppers are bastards' or ‘1488' was obviously a very common one for the numerical code of Neo-Nazi references.” (Interviewee 1, May 2024)^21^

Not only are young people exposed to extreme messages on mainstream social media but also—and as mentioned by Interviewee 1 above—young people can be involved in regular communication with extremist actors taking place in gaming (adjacent) spaces where difficulties related to moderation are evident, i.e. real time verbal communication and use of symbols, as considered above. When discussing these issues, some interviewees appeared to be very disappointed in the gaming developers, as they “have a philosophical preference for privacy even at the cost of safety,” allowing for complete anonymity of users (Interviewee 8, June 2024).^22^ The same participant also pointed out the fact that “games have this really unique characteristic of they have public matchmaking with strangers… they have an opportunity for rapid rapport building,” which again can be quite problematic, especially when exposing young users to a wider cohort of unknown individuals and where extremist actors can also lurk.

#### 4.3.3 What practitioners can do

There was, however, hope among the moderators, P/CVE practitioners, and the experts interviewed that certain tried-and-tested prevention tactics and counter-response techniques do work in the gaming-adjacent space. One key technique touched upon—at the preventative level—was improving content moderation by raising awareness about harmful online content and behaviors.

“We can't police all these platforms…so our job as frontline practitioners would be to make sure teachers, pupils, and parents are aware of some of these risks and what goes on some of these platforms. By raising that awareness, hopefully, responsible parents will have those conversations with their children, and teachers keep an eye out for it, the conversations they might hear in the classroom.” (Interviewee 1, May 2024)^23^

Participants acknowledged the impossibility of policing all platforms effectively and real-time speech for all the issues discussed above. However, as stressed by Interviewee 1, they highlighted the need to raise awareness among young people's trusted adults (such as their parents and teachers) on the risks connected to extremism in gaming-adjacent spaces. In so doing, the policing effort can be strengthened by offline inputs from families and schools. Awareness raising also appeared to be essential to educate users themselves (using age-appropriate tools and conversations) to be able to transform them into active bystanders confident enough to report something that “does not look right” on gaming-adjacent platforms (Moonshot Team, 2022).

Another tool mentioned by the interviewees at the preventative level related to the use of counter-narratives. The prime aim here—expressed by respondents—was to educate users about the dangers of extremist content, using video games and adjacent platforms as an online outreach tool to reach individuals at risk of radicalization. However, interviewees disagreed about how impactful these narratives truly are and whether they would be sufficiently proactive in turning the tide on extremism—something that is not globally prohibited in all platform terms of service.

Another aspect of counter-response efforts touched upon by respondents was the harnessing of artificial intelligence (AI) for content moderation and policing purposes on gaming-adjacent platforms. In the past, AI has proven useful in identifying and flagging problematic textual content, but limitations exist, particularly in nuanced online spaces like gaming, where video and audio content are germane:

“People might just be talking about Call of Duty, and they will be talking about weapons and attacks. They might even say I'm going to kill you. What they mean is that I'm gonna kill you in the game… so I think this is very difficult for automated detection.” (Interviewee 12, July 2024)^24^

Besides the difficulties related to moderating in the context of specific games and their explicit reference to violence and killing (as also discussed in the section above), participants suggested that bots might be a potentially fruitful avenue, capable of issuing automatic bans for posts with harmful language and providing some (light) relief for human content moderators. Participants explained that bots operate based on a set list of words, which they detect and ban. Some platforms (like Facebook) are already progressing in the use of bots, training them to identify graphic violence and hate speech (Facebook, n.d.). However, key pitfalls were also identified, including how bots fall short in detecting sarcasm or masked language, allowing certain content to slip through, and the ultimate need for human content moderation in the most sensitive and trickiest of cases.

“You can set up a banned word list, and we'd constantly review that to find new terms and symbols, because people would find ways around it…also, at the end of the day, you need that human to read the content to see if there is any sarcasm in it.” (Interviewee 5, June 2024)^25^

“Some platforms like Steam use auto moderation, so they blur out certain terms, and you can just see little hearts that appear instead of the term. The problem is you can still search for those terms…so if I search for the N-word I will get posts that feature the N word and they appear blurred out, but everybody knows what is being said here, so the problem remains.” (Interviewee 6, May 2024)^26^

As highlighted by both interviewees 5 and 6, while AI-based automatic content moderation is useful to perform basic tasks (e.g., detecting and banning a series of words), it has its limits, and harmful content often slips through. Hence, in order to perform efficient content moderation on gaming-adjacent platforms, human moderation is essential. However, for the most severe and pressing cases, interviewees stressed that threat escalation protocols would be very useful in assisting human policing efforts. Creating precise protocols that automatically identify direct threats to life or other kinds of specific threats with a timeline attached (or “where people would encourage harassment of specific streamers or individuals associated with streamers”) (Interviewee 6, May 2024).^27^ Such protocols would also be used when users use games to enact violent fantasies and then clearly express how much they enjoy committing violence against certain outgroups in the game.

While participants acknowledged that in some cases such statements do not necessarily reflect intent to perform an act that is criminal in nature, “it reflects quite disturbing views and disturbing propensities of the user” that might need further preventative efforts and interventions for it not to escalate to a problematic offline case (Interviewee 6, May 2024).^28^ Some of the most common online actions indicated by such views include coordinated online harassment campaigns, such as raids that these users will engage in to drive users they disagree with off other platforms. After discussing the use of threat escalation protocols, interviewees argued that fostering better partnerships with law enforcement to make platforms safer is pivotal.

“Law enforcement agents should create accounts and understand how the platforms operate…we need to move beyond people of a certain age only having a Facebook account…everyone needs to be a little bit more fluent and demystify any platform they think might be a problem.” (Interviewee 2, June 2024)^29^

As stated by interviewee 2, it was noted that law enforcement agents need to better understand how the platforms and their subcultures operate in order to interdict them better. Additionally, in order to sustain content moderation on such platforms, it was noted that providing better mental health support and relief for moderators was essential, as continual exposure to harmful content was proven to have a detrimental impact on their wellbeing in the long term.

Finally, the interviewees deemed that gaming-adjacent platforms could enhance and strengthen these efforts by increasing transparency regarding content moderation guidelines and the enforcement of accountability measures. In order to foster greater confidence, interviewees stressed that content moderation guidelines should be made more transparent and that platforms should hold both users and themselves accountable under their terms of service, enabling more consistent and effective enforcement of such sanctions. Moreover, the stressing of legal ramifications for users who breach these terms of service is also essential in reinforcing responsible online behavior and acts as a deterrent for potentially malicious acts in the future.

The most worrying aspect of this, as reported by moderators, was recidivism and threats against moderators themselves: “…there are like some lines where I'm like, why would this user be allowed back? You know, like the user that sets me like as an agent, that Threats and stuff like that. I don't feel that they should ever be allowed back on the platform, but they will be. Eventually, they will be. It's just a few months, and they're gonna be back” (Interviewee 5, June 2024).^30^

## 5 Discussion

This study contributes to the expanding literature on the intersection of gaming and extremism by focusing on a relatively underexplored aspect: the exploitation of gaming-adjacent platforms for extremist purposes and the systemic and practical challenges in moderating such spaces. The findings offer a nuanced picture of how extremist ideologies permeate these platforms, not merely as passive content but as part of interactive and social dynamics that can facilitate radicalization, recruitment, and the normalization of harmful worldviews.

One of the most pressing insights from the study is the difficulty in distinguishing between extremist content and outright illegal activity. The co-mingling of hate speech, child sexual abuse material (CSAM), glorification of violence, and fringe ideological content points to a broader issue: gaming-adjacent platforms operate in gray areas where the enforcement of Terms of Service (ToS) and legal thresholds often do not align (Citron, 2014; Conway et al., 2019). This ambiguity presents major challenges for content moderators who must make rapid and consequential decisions, often without clear precedent or adequate institutional support (Roberts, 2019).

Unlike earlier models of radicalization that emphasized structured group involvement (McCauley and Moskalenko, 2008), this study underscores the post-organizational, networked, and often self-directed nature of radicalization in gaming-adjacent spaces. The symbolic adoption of extremist memes, slogans, and avatars enables users to signal their affiliation with ideological subcultures without formal affiliation (Ebner, 2020; Koehler, 2014). These dynamics highlight a critical need to rethink how radicalization is conceptualized in the digital age—not as a linear process but as a fluid set of interactions shaped by algorithms, anonymity, and cultural immersion (Valentini et al., 2020). Moreover, this study supports growing evidence that gaming-adjacent platforms act as bridges between normative digital spaces and high-risk ecosystems. The practice of content funneling—where initial interactions take place in general-interest gaming communities before moving to more extremist spaces—echoes recent digital ethnographic research on right-wing extremist use of Discord and Steam (Winkler et al., 2024; Awan, 2017).

The findings also reveal a striking disconnect between the affordances of these platforms and their preparedness to handle extremist misuse. While platforms like Discord and Twitch have made strides in content moderation, responses remain largely reactive and inconsistent (Crawford and Gillespie, 2016). AI tools, while useful for flagging textual content (Maras and Alexandrou, 2019), often fall short in video and audio-heavy environments such as gaming, where coded language, memes, and sarcasm are prevalent (Gillespie, 2018). Moreover, the mental health toll on human moderators who are exposed to disturbing content for extended periods has been well-documented (Roberts, 2019). Without systemic support, moderation becomes not only ineffective but also unsustainable—particularly given the increasing complexity of identifying borderline content and balancing freedom of expression with safety.

Finally, while there is growing interest in using gaming-adjacent platforms as tools for countering violent extremism (P/CVE), this study highlights both the promise and the limitations of these efforts. Counter-narrative campaigns can be effective (Briggs and Feve, 2013) but must be deeply embedded within the vernacular and culture of gaming communities to resonate (Davies et al., 2016). There was notable skepticism among practitioners about the efficacy of these interventions in isolation, particularly given that extremist influencers are adept at co-opting platform affordances to maximize their reach and appeal (Conway, 2017). The respondents within this study advocated for a more cultural approach to P/CVE—one that addresses the toxic norms and hyper-masculinity prevalent in some gaming environments. These cultural features, if left unchecked, can serve as fertile ground for extremist recruitment and radicalization (Bezio, 2018; Frissen et al., 2015).

## 6 Conclusion

To conclude, this article presents the first comprehensive analysis of how policing communities have been attempting to tackle and interdict extremist content on gaming-adjacent platforms. Extremists have been exploiting these platforms for a while (c. 8 years)—using them for radicalization, recruitment, and the dissemination of their messages (Davey, 2024). As has been alluded to in this article, gaming-adjacent platforms exist within a broader ecosystem of emerging technologies, where extremist actors engage in forms of “opportunistic pragmatism,” circumventing codes of practice and using these platforms to funnel individuals into more closed and concealed online spaces (Davey and Ebner, 2017).

Utilizing 13 semi-structured expert interviews with content moderators, P/CVE practitioners, and academic and technology industry experts, this article has attempted to plumb the depths of what extremist activism looks like on these platforms, the issues involved, and how online tech companies, civil society, and state bodies have attempted to engage with such content. Like mainstream social media platforms 10 years ago, gaming-adjacent platforms are in the early stages of interdicting such content, but only in the most harmful of circumstances. Therefore, more needs to be done to interdict extremist content. Legislation such as the EU's Digital Services Act or the UK's Online Safety Act is a step in the right direction. These will require tech platforms, including gaming-adjacent platforms, to remove extremist (or otherwise harmful) content if flagged by relevant national authorities. However, these new laws are reactive, not proactive. They respond to instances of (violent) extremism once they have already occurred, not while in process.

As was noted by interviewees, therefore, more effort needs to be exerted on proactive enforcement and precise threat escalation procedures. In addition, more transparency is needed regarding content moderation guidelines and the accountability measures used in their enforcement. The most worrying aspect of enforcement attempts reported by moderators was recidivism and threats against moderators themselves, where repeat offenders were allowed back onto platforms without facing repercussions, thereby enabling them to perpetrate attacks again.

Importantly, however, we unearthed a picture of a suite of potential P/CVE efforts. This included certain tried-and-tested prevention tactics and counter-response techniques that have proven effective in the gaming-adjacent space, such as awareness-raising campaigns, counter-narratives, automated AI bots, and enhanced partnerships with law enforcement. Therefore, whilst we wait for stronger measures against awful but lawful content at the platform level and at scale, a more bottom-up approach needs to be adopted when it comes to P/CVE and attacking the cultures that lead to extremists to thrive on such platforms in the first place (Hartgers and Leidig, 2023).

### 6.1 Limitations

Nonetheless, several limitations in this study should be acknowledged. The relatively small sample size of 13 experts, while providing rich insights, may not capture the full diversity of experiences across the vast ecosystem of gaming-adjacent platforms. Furthermore, the focus on Twitch, Steam, and Discord, while significant, overlooks other emerging platforms, such as DLive, Odysee, and Trovo, where extremist activity may also be present but remains underexplored. Given the rapidly evolving nature of extremist tactics and the continual emergence of new platforms, the findings may also quickly become outdated as actors adapt to new environments and tools—though this is always a hazard endemic in online research.

## 7 Future research

Future research should therefore broaden its scope to encompass a wider array of gaming-adjacent platforms, incorporating longitudinal studies that track changes in extremist content and the effectiveness of moderation over time. A deeper examination of user behavior within these spaces could also shed light on the processes of radicalization and the ways extremist ideologies propagate in gaming communities. Such insights would be invaluable in crafting more nuanced and effective interventions.

### 7.1 Implications

The implications of this research are wide-ranging. Policymakers must continue to develop clear and consistent frameworks that hold platforms accountable while striking a balance between free expression and harm prevention. Tech companies in the gaming sector should invest in advanced moderation technologies and comprehensive staff training, including the deployment of AI systems capable of detecting subtler forms of extremist content. Equally, community engagement initiatives that foster positive online cultures and raise awareness of extremist risks are critical to building resilience from the ground up.

In summary, while legislative and technological measures form an essential part of the response to extremism on gaming-adjacent platforms, addressing the root causes and cultural drivers of extremist activity demands a multi-layered, collaborative approach that spans regulators, industry, civil society, and gaming communities alike.

### 7.2 Recommendations

As a result of our findings and discussion, our recommendations call for coordinated action among platforms, regulators, researchers, and practitioners to address the complex challenges surrounding radicalization and harmful content within gaming environments. These recommendations carry both practical and theoretical implications that must be carefully considered.

First, platforms and regulators are urged to intensify efforts in monitoring and managing content that, while technically lawful, can still be harmful. Practically, this involves strengthening moderation systems—both algorithmic and human-led—to more effectively identify and address such content (Gillespie, 2018). It also requires updating platform policies and regulatory frameworks to better capture and mitigate the risks posed by this gray area of content (Global Internet Forum to Counter Terrorism, 2023). In the absence of adequate top-down regulation, a bottom-up strategy should also be implemented. Theoretically, this approach necessitates a shift from a purely legalistic understanding of online harm to one centered on broader notions of social and psychological harm (Farrand, 2024; Kalliris, 2024). Such a model proposes new forms of governance based on user participation, signaling a shift toward distributed or hybrid models of content moderation (Kira, 2025). Furthermore, it demands a rethinking of digital citizenship, where users are not passive consumers but also co-creators of safer online spaces (Estellés and Doyle, 2025).

For researchers, there is a strong call to rethink traditional methodological approaches to studying radicalization in gaming. On a practical level, researchers are encouraged to adopt more active and immersive methodologies—such as digital ethnography, participant observation, and cross-platform content analysis—that go beyond passive techniques like surveys or automated NLP tools (Hutchinson, 2023; Cheah, 2025). This shift also entails fostering interdisciplinary collaboration across game studies, sociology, psychology, and data science to adequately address the multifaceted nature of radicalization within gaming environments. Theoretically, this necessitates a more holistic, ecosystem-oriented approach that moves beyond individual platform dynamics and instead maps how radicalization flows across interconnected digital spaces (Conway et al., 2019). It critiques the limitations of purely computational models and calls for deeper engagement with the cultural and emotional contexts in which content is consumed and produced (Döveling et al., 2018). Researchers are thus encouraged to investigate the interplay between online behaviors and offline identities, community norms, and platform affordances (Lüders et al., 2022).

Finally, practitioners and policymakers are encouraged to recognize the dual role of gaming platforms as both potential risk zones and powerful tools for intervention. Practically, this includes designing platforms with safety in mind—through features such as content filtering, community moderation systems, and behavioral nudges (Donabauer et al., 2024). Equally important is fostering inclusive, diverse digital communities that counteract toxic subcultures and prevent them from becoming incubators of radicalization (Schlegel, 2021a,b). These platforms also offer untapped potential for positive outreach to individuals at risk by engaging them through interactive storytelling, peer mentorship, and prosocial gaming initiatives (RAN Practitioners, 2023). On a theoretical level, these strategies highlight the criminogenic potential of online environments, where toxic subcultures may serve as precursors to more extreme ideologies (Hartgers and Leidig, 2023; Ebner, 2020). This underscores the importance of understanding “extremist-adjacent” behaviors as part of a radicalization continuum rather than isolated anomalies. It also suggests the need for behavioral precursor models that identify early signals of vulnerability or ideological drift (Davey and Ebner, 2017). More broadly, this framing supports the conceptualization of gaming platforms as *dual-use spaces*—sites that may either contribute to or help prevent radicalization, depending on how they are governed, designed, and culturally curated. In summary, we advocate for a multi-layered response that combines regulatory oversight, community empowerment, active research, and inclusive design.

## Data Availability

The datasets presented in this article are not readily available because not for public release. Requests to access the datasets should be directed to william.allchorn@aru.ac.uk.

## Footnotes

^1^Twitch (https://www.Twitch.tv/) is a major platform that can be considered gaming adjacent. Twitch is a wildly popular livestreaming site—it had 31 million daily active users as of March 2023 (Ruby, 2023) and is primarily used to broadcast gaming to a global audience. Although the streaming of games makes up the majority of its activity, it is also used for broader communication with popular streamers through its “just chatting” feature (Hutchinson, 2020).

^2^Steam (https://store.steampowered.com/) is, at its core, a video game distribution service. Launched in September 2003 as a software client for Valve's games, it expanded in 2005 to distribute third-party titles. However, since its launch, it has introduced a range of other functionalities, including social networking and community building. Accordingly, Steam can be seen to be both a gaming platform and a gaming-adjacent platform.

^3^The most well-known gaming-adjacent platform is perhaps Discord (https://Discord.com/). Discord is a chat platform that allows users to communicate with instant messaging, video, and chat calls and was originally created to facilitate communication between gamers. One key function of Discord is the ability for users to create “‘servers”—spaces in which groups of individuals can communicate and build communities.

^4^In addition to Twitch, there is a range of other less popular alternative livestreaming platforms, including DLive (https://DLive.tv/) and Bigo (https://www.bigo.tv/), also exploited by extremist actors, especially within the far-right ideological milieu.

^5^Interviewee 12 (June 2024) *Interview with William Allchorn*. Online. Academic Expert. MS Teams.

^6^Interviewee 11 (June 2024) *Interview with William Allchorn*. Tech Industry Expert. Online. MS Teams.

^7^Interviewee 4 (June 2024) *Interview with Elisa Orofino*. Content Moderator. Online. MS Teams.

^8^Interviewee 5 (June 2024) *Interview with William Allchorn*. Content Moderator. Online. MS Teams.

^9^Interviewee 6 (May 2024) *Interview with William Allchorn*. P/CVE Practitioner. Online. MS Teams.

^10^Interviewee 2 (June 2024) *Interview with Elisa Orofino*. P/CVE Practitioner. Online. MS Teams.

^11^Interviewee 8 (June 2024) *Interview with William Allchorn*. Tech Industry Expert. Online. MS Teams.

^12^Interviewee 3 (May 2024) *Interview with William Allchorn*. Academic Expert. Online. MS Teams.

^13^See text footnote 10.

^14^Interviewee 1 (May 2024) *Interview with William Allchorn*. P/CVE Practitioner. Online. MS Teams.

^15^Interviewee 13 (May 2024) *Interview with Elisa Orofino & William Allchorn*. Tech Industry Expert. Online. MS Teams.

^16^Interviewee 12 (July 2024) *Interview with William Allchorn*. Online. Tech Industry Expert. MS Teams.

^17^See text footnote 16.

^18^Interviewee 5 (June 2024) *Interview with Elisa Orofino*. Content Moderator. Online. MS Teams.

^19^Interviewee 13 (May 2024) *Interview with Elisa Orofino & William Allchorn*. Content Moderator. Online. MS Teams.

^20^See text footnote 12.

^21^See text footnote 14.

^22^See text footnote 11.

^23^See text footnote 14.

^24^Interviewee 12 (July 2024) *Interview with William Allchorn*. Academic Expert. Online. MS Teams.

^25^See text footnote 18.

^26^See text footnote 9.

^27^Ibid.

^28^bid.

^29^See text footnote 10.

^30^See text footnote 18.
